# The Burden of Maternal Psychological Distress in Pregnancy: Impact of Antepartum Risk Factors in a Pakistani Tertiary Care Setting

**DOI:** 10.1192/j.eurpsy.2026.10745

**Published:** 2026-07-31

**Authors:** Z. Farooq, R. Tahir, S. Mansoor, M. Ali, N. Bibi

**Affiliations:** 1 Phoenix Care Centre, HSE North Dulin City Mental Health Services, Dublin, Ireland; 2 Social Sciences, International Islamic University Islamabad, Islamabad; 3 Psychiatry, Fauji Foundation Hospital, Rawalpindi, Pakistan; 4 Adult Community Mental Health Services, HSE North Dublin Mental Health Services, Dublin, Ireland; 5 Social Sciences, Virtual University of Pakistan, Islamabad, Pakistan

## Abstract

**Introduction:**

Antenatal psychological morbidities, encompassing depression, anxiety, and stress, are pervasive yet frequently underrecognized, particularly in low- and middle-income countries. These conditions jeopardize maternal well-being and fetal outcomes, accentuating the imperative for timely detection and intervention

**Objectives:**

This study aimed to delineate the prevalence of depression, anxiety, and stress among pregnant women and elucidate their associations with gestational age, obstetric complications, and pregnancy intentionality.

**Methods:**

A cross-sectional analysis was conducted on 200 pregnant women attending antenatal clinics. Psychological outcomes were measured using the Urdu-adapted Depression, Anxiety, and Stress Scale (DASS-21), demonstrating acceptable internal consistency (Cronbach’s alpha: depression > 0.70; anxiety = 0.776; stress = 0.821). Associations between mental health indices, gestational age, obstetric complications, and pregnancy intent were evaluated via chi-square tests.

**Results:**

Depression was observed in 46% of participants, with 28.5% manifesting moderate and 3% exhibiting extremely severe symptomatology. Depression correlated significantly with obstetric complications (χ² = 126.218, p < .001), particularly histories of abortion and hypertension, and with gestational age (χ² = 32.998, p < .001), peaking in the third trimester. Anxiety and stress exhibited analogous associations with complications (anxiety χ² = 152.274; stress χ² = 134.864; p < .001) and trimester (anxiety χ² = 45.551; stress χ² = 65.183; p < .001). Women with obstetric complications, anemia, or hypertension reported heightened psychological distress, particularly in advanced gestation. Paradoxically, planned pregnancies were linked with more severe depression (χ² = 12.747, p = .005), whereas stress severity was not significantly modulated by pregnancy intentionality.

**Conclusions:**

Approximately half of pregnant women experienced depressive symptomatology, with one-third attaining clinically significant severity. Psychological morbidity was strongly associated with advancing gestation and adverse obstetric history. The disproportionate burden of extreme depression among women with planned pregnancies may reflect sociocultural imperatives, including gendered expectations. These findings underscore the necessity for systematic antenatal mental health screening and culturally attuned psychosocial interventions targeting medically and socially vulnerable populations.

**Disclosure of Interest:**

None Declared

